# Translational selection on SHH genes

**DOI:** 10.1590/S1415-47572010005000035

**Published:** 2010-06-01

**Authors:** Mohammadreza Hajjari, Behnaz Saffar, Atefeh Khoshnevisan

**Affiliations:** 1 Department of Genetics, Faculty of Biological Science, Tarbiat Modares University, TehranIran; 2 Department of Genetics, Faculty of Science, Shahrekord University, ShahrekordIran; 3 Biotechnology Research Department, Shahrekord University, ShahrekordIran

**Keywords:** bioinformatics, codon usage bias, correlation, translational selection

## Abstract

Codon usage bias has been observed in various organisms. In this study, the correlation between SHH genes expression in some tissues and codon usage features was analyzed by bioinformatics. We found that translational selection may act on compositional features of this set of genes.

Synonymous codons are codons which code for the same amino acid. Non-random usage of these codons is a widespread occurrence which has been observed in many organisms such as *Drosophila melanogaster, E.coli* and *Saccharomyces cerevisiae* (Sharp and Li, 1987; Ikemura, 1985; Powell and Moriyama, 1997). One of the models that express such biases is translational selection (Zuckerkandl and Pauling, 1965; Ikemura, 1981; Sørensen *et al.*, 1989; Debry and Marzluff, 1994; Levy *et al.*, 1996). Correlations between synonymous codon usage and gene expression levels are usually attributed to the higher abundance of isoaccepting tRNA for optimal codons to maximize translational efficiency both in unicellular (Dong *et al.*, 1996; Rocha, 2004) and multicellular organisms (Moriyama *et al.*, 1997; Duret, 2000; Kanaya *et al.*, 2001; Lavner and Kotlar, 2005). In vertebrates there has been much debate on this correlation (Wolfe and Sharp, 1993; Musto *et al.*, 2001; Konu and Li, 2002). Moreover, this correlation is not clear in mammals including human beings.

Thus, analyzing codon usage bias in specific tissues and specific subsets of genes is an interesting area of investigation. The Sonic Hedgehog (SHH) pathway is one of the most important developmental pathways which is conserved from flies to human. The proteins that are involved in this pathway are not only critical in embryonic development but have also been implicated in certain cancers. In this study we analyzed the correlation between synonymous codon usage features of SHH pathway genes and their expression in human tissues. The results are expected to provide valuable information on translational efficiency of this pathway in humans. By such information, analyzing cancer mechanisms and causative mutations may become more expedient.

SHH pathway genes were collected from a Hedgehog Signaling Pathway Database. In total 31 genes were selected from SHH signal receiving cells. The NCBI database was used to obtain their CDSs. To avoid statistical errors, the CDSs were aligned by ClustalW. Similarity scores of every two genes were between one and 68. Normalized expression levels in the brain, embryonic tissue, prostate, ovary, testis, liver, muscle, and the eye were retreived from the SOURCE database. By using the FREQSQ program we calculated synonymous codon usage features and percentage of each synonymous codon in each codon family that codes for the same amino acid. Statistical analysis was performed by using the MINITAB13 program. Codons with p values below 0.01 were considered as significant features.

After analyzing 59 synonymous codon usage features (all codons except termination codons, Methionine, and Tryptophan codons) for 31 genes in the SHH pathway, 1829 features were obtained. Then, by analyzing the relationship between gene expression levels in eight tissues and synonymous codon usage features, 13 significant features were noted in four tissues (brain, ovary, testis and the eye) (Table 1). Among significant features, three of “synonymous codon usage” were shared by two tissues. Regression equations were then obtained for significant features (Table 2). For one amino acid (L) we could find two different and significant codon usage features in brain and testis.

The above analysis revealed a correlation between synonymous codon usage and expression level of SHH genes in brain, testis, ovary, and the eye. But this correlation was not observed in embryonic tissue, prostate, liver, and muscle. Thus, translational selection may select synonymous codons in genome sequences. Our analysis indicates that SHH gene expression may be regulated at a post-transcriptional level. Common features between two tissues support the assumption that the same mechanism may act in common pathways. For instance, codon usage bias can be attributed to the frequency of isoaccepting tRNAs in a tissue. This may also account for the herein observed correlations, because differences in relative tRNA abundance with a maximum range of tenfold variation have been detected in different human tissues (Ditmar *et al.*, 2006). Furthermore, CTA and TTA, which encode leucin, are usage-biased in the brain and testis. This result indicates the criticality of this amino acid and its tRNAs in the two tissues. There are no significant features for synonymous codon usage in prostate, embryonic tissues, liver, and muscles, wherefore translational selection in these tissues may be absent

The average expression in brain and testis was higher than in other tissues. This result indicates translational efficiency and accuracy. In fact, the evolution of codon bias in highly expressed genes is hypothesized to be a result of natural selection for increased protein elongation rates (Bulmer, 1999) or minimized errors in mRNA translation (Akashi, 1994). Furthermore, the frequency of tRNAs or factors which are involved in translation may be different in various tissues. This result has notable implications for understanding the molecular mechanisms of tissue development and cancer. It is important to mention that other compositional features may also affect gene expression in selected tissues. Appropriate analysis can help in understanding molecular mechanisms of gene expression and mRNA translation. Hence, the relationships between genome, transcriptome and proteome may become clearer.

## Figures and Tables

**Table 1:** Significant features for the correlation between expression levels of SHH genes and synonymous codon usage features in human tissues.

Tissue	Number of expressed genes	Average expression	Highest expressed gene	p value	*r*_s_ value	Feature
Brain	31	63.84	PRECAKB	0.001	0.548	CTA(L)
				0.002	0.542	TGT(C)*
				0.002	0.542	TGC(C)*
				0.002	0.542	CCA(P)*

Ovary	21	3.905	GPC1	0.008	-0.563	ATA(I)
				0.004	0.603	CAC(H)
				0.004	-0.606	GGA(G)

Testis	28	15.43	IFT88	0	0.647	TTA(L)
				0.004	0.522	CCA(P)*
				0.002	0.561	ACT(T)
				0.005	0.519	TGT(C)*
				0.005	-0.519	TGC(C)*

Prostate	24	5.304	CSNK1A	-	-	-

Embryonic tissue	22	5.45	GPC4	-	-	-

Eye	26	6.231	GSK3A and PRECAKB	0.008	0.509	TCA(S)

Liver	20	6.65	CSNK1A	-	-	-

Muscle	26	7.375	GLI1	-	-	-

*: Common features.

**Table 2:** Regression equations for significant features.

Tissue	Equation
Brain (4 significant features)	EXP = - 49.9 + 13.7 CTA(L)
	EXP = - 75.6 + 2.79 TGT(C)
	EXP = 203-2.79 TGC(C)
	EXP = - 89.3 + 5.26 CCA(P)

Ovary (3significant features)	EXP = 6.68-0.187 ATA(I)
	EXP = - 1.34 + 0.0954 CAC(H)
	EXP = 8.57-0.165 GGA(G)

Testis (5significant features)	EXP = 3.54 + 1.28 TTA(L)
	EXP = - 5.19 + 0.671 CCA(P)
	EXP = - 13.4 + 1.03 ACT(T)
	EXP = - 3.02 + 0.348 TGT(C)
	EXP = 31.8-0.348 TGC(C)

Eye (1significant feature)	EXP = 1.45 + 0.310 TCA(S)
